# Remediation of Cr(VI) Polluted Groundwater Using Zero-Valent Iron Composites: Preparation, Modification, Mechanisms, and Environmental Implications

**DOI:** 10.3390/molecules29235697

**Published:** 2024-12-02

**Authors:** Manyu Yang, Xueyan Zhang, Yongchang Sun

**Affiliations:** 1 Key Laboratory of Subsurface Hydrology and Ecological Effects in Arid Region, Ministry of Education, Chang’an University, Xi’an 710054, China; yangmanyu1216@163.com (M.Y.); qq811877630@gmail.com (X.Z.); 2 Key Laboratory of Eco-Hydrology and Water Security in Arid and Semi-Arid Regions of Ministry of Water Resources, Chang’an University, Xi’an 710054, China; 3 Department of Environmental Engineering, School of Water and Environment, Chang’an University, Xi’an 710054, China

**Keywords:** nZVI, groundwater, Cr(VI), mechanism, toxicity

## Abstract

The extensive application of chromium (Cr) in many industries has inevitably resulted in the release of Cr(VI) into the groundwater environment, thus posing damage to the ecosystem and human health. Nano zero-valent iron (nZVI) has been widely studied and applied in the remediation of Cr(VI)-contaminated water as an ideal material with high reductive capacity, which enables the transformation of teratogenic and carcinogenic Cr(VI) into less toxic Cr(III). This review comprehensively summarizes the preparation and modification methods of nZVI Cr(VI) removal performance and mechanisms by nZVI and modified nZVI materials. The field applications of nZVI-based materials, such as combining the injection well and the permeable reactive barrier (PRB) to remove Cr(VI) in groundwater, have been reported. Subsequently, the potential toxicity of nZVI-based materials to organisms during environmental application has been highlighted in the current study. Finally, the review outlines potential improvements and explores future directions for the use of nZVI-based materials in groundwater contamination remediation.

## 1. Introduction

Water plays a crucial role in promoting urbanization, preserving the natural ecosystem, and sustaining life [1]. With the ongoing economic and industrial development, the ever-increasing demand for water resources has led to increased dependence on groundwater, which is regarded as an important source of drinking water and irrigation water [2]. According to studies, groundwater provides drinking water for approximately one-third of the global population [3], and 40% of the cultivated land depends on groundwater for irrigation, either partially or entirely. Based on previous studies, 80% of the shallow groundwater and 30% of the deep groundwater in China have been contaminated to varying extents [4]. Consequently, the protection and governance of groundwater have become urgent priorities.

Chromium (Cr) is widely utilized in metallurgy, tanning, dyeing, electroplating, and other industries [5] due to its excellent properties in hardness, corrosion resistance, and gloss. However, leakage, poor storage, improper disposal, and careless management of industrial facilities are major contributors to Cr contamination in groundwater, particularly in industrial zones. It was reported that in 2020, the concentration of total Cr in groundwater near an industrial zone in India reached 140 mg/L [6], significantly exceeding the allowable limit of 0.05 mg/L for chromium stipulated by the World Health Organization (WHO). The groundwater around an abandoned chromium slag site in southern China is contaminated with chromium over an area of about 40,000 m^2^, and the contaminated area is at risk of spreading further [7]. In addition, wastewater irrigation of farmland, which occurs mostly in water-scarce areas, can also lead to the accumulation of heavy metals in groundwater. The concurrent occurrence of irrigation and groundwater exploitation further enhances the transport of chromium from soil to deep groundwater [8]. Most heavy metals possess toxicity, non-biodegradability, bioaccumulation, trophic magnification, and other geochemical characteristics [9], and Cr is no exception. Chronic exposure to Cr, whether through the skin, digestive system, or respiratory tract, can result in a range of health issues, including dermatitis, chronic ulcers, rhinitis, nasal perforation, liver and kidney dysfunction, cancer, or even death [10].

In the natural environment, Cr mainly exists in two stable states, trivalent chromium (Cr(III)) and hexavalent chromium (Cr(VI)) [11]. Cr(III) is less toxic and usually exists in the precipitated form (e.g., chromium hydroxide). Cr(VI), on the contrary, with high solubility and strong mobility [12], is mainly available as chromate (CrO_4_^2−^) or dichromate (Cr_2_O_7_^2−^). Reducing Cr(VI) to Cr(III), followed by in situ precipitation, is currently the most extensively researched remediation strategy for Cr(VI) pollution. Nano zero-valent iron (nZVI), a novel environmental remediation material, possesses strong reduction and adsorption capabilities, allowing it to effectively remove a wide range of pollutants, including antibiotics [13,14], iodinated contrast media (ICM) [15], organic dyes [16,17], pesticides [18], inorganic anions [19], heavy metals [20], etc. Characterized by low cost, easy recycling, and environmental friendliness, nZVI has been one of the most intensively researched environmental clean-up materials over the past two decades [21].

However, the application of bare nZVI in contaminated sites is limited due to its high surface energy, easy aggregation, and passivation [22]. To maximize the advantages of nZVI, researchers have developed various countermeasures. For example, the small particle size of nZVI allows it to be embedded in porous structures of support materials, such as biochar, chitosan, zeolite, etc., effectively preventing their agglomeration [23]. A variety of stabilizers, including anionic or non-ionic surfactants and high polymers, can effectively prevent nZVI agglomeration and enhance mobility [24,25]. Furthermore, strategies such as doping with second metals (e.g., Ag, Ni, and Pd) [26] and modifying nZVI with persulfate (PS) have been employed to improve pollutant removal efficiency by facilitating electron transfer from nZVI. Recent studies have also demonstrated that the combination of multiple modification techniques can further enhance the performance of nZVI.

In parallel to the research on the modification of nZVI on the laboratory scale, the application and monitoring of nZVI-based materials in the contamination site and the potential toxicity to organisms and the environment are also big issues to be taken seriously. nZVI is by far the most widely used NP in remediation. nZVI-based materials have been shown to effectively remove Cr(VI) through direct groundwater injection techniques and permeable reaction wall (PRB) techniques. Although the unique characteristics of nZVI bring beneficial application prospects, it is rarely used in the field. This is likely due to the uncertainty caused by the lack of field data on the use of nZVI for the remediation of contaminated sites and the study of the behavior, fate, and toxicity of nZVI particles in the environment.

In this paper, the preparation of nZVI and nZVI-based materials and the progress of Cr(VI) removal are discussed. It also provides an overview of the latest developments in the field application of nZVI. Additionally, the potentially toxic effects of nZVI on organisms (like plants, humans, animals, and microorganisms) are briefly discussed.

## 2. Nano Zero-Valent Iron (nZVI)

### 2.1. Structures and Properties of nZVI

nZVI is 1–100 nm in size and exhibits a core-shell structure [27]. The center of a single nZVI molecule was Fe^0^, and the surface contained iron oxides such as FeO, Fe_2_O_3_, Fe_3_O_4_, and iron hydroxides because of the oxidation of metallic iron [28]. It is the core that provides the reducing power (the electron source) for the reactions, whereas the shell provides the site for chemically complex reactions (chemisorptions) and electrostatic interactions [29]. The defective and disordered nature of the oxide shell makes it potentially more active, and the core-shell structure has important implications for the chemical properties of nZVI. The interaction mechanism of Cr(VI) by nZVI is mainly attributed to reducing chromium to the less toxic trivalent chromium. The possible reactions are as follows: Equations (1) and (2) [30].

2HCrO_4_^−^ + 3Fe^0^ + 14H^+^ → 3Fe^2+^ + 2Cr^3+^ +8H_2_O (1)

Cr_2_O_7_^2−^ + 6Fe^2+^ + 14H^+^ → 6Fe^3+^ + 2Cr^3+^ + 7H_2_O (2)

### 2.2. Preparation Methods and Procedures of nZVI

The preparation technology of nZVI is relatively mature. It can be synthesized via physical and chemical methods. Physical methods include mechanical ball milling, vapor deposition method, and ultrasound-assisted processes, whereas chemical methods include liquid-phase reduction, microemulsion method, pyrolysis iron pentacarbonyl, electrochemical method, and green synthesis. The different methods and corresponding processes, advantages, and disadvantages of the synthesis of nZVI are summarized in Table 1. In the physical method, the shape and size of nZVI particles can be well controlled by adjusting the process parameters, and the usage of a higher amount of chemicals is strictly avoided. The purity of nZVI prepared by the physical method is usually high, but so are the corresponding costs and equipment requirements [31,32]. Using the microemulsion method can control the shape and diameter distribution of nZVI through simple operations, but the cost is high, and secondary pollution would be caused [33]. Instead, liquid-phase reduction with sodium borohydride (NaBH_4_) is considered to be the simplest and most widely used method for preparing nZVI, allowing for the easy preparation of fresh, highly active nZVI in the laboratory and on the construction site. The synthetic routes of nZVI involve the reduction of Fe^2+^/Fe^3+^ salt solution with NaBH_4_ as a reducing agent. However, the high price and potential toxicity of NaBH_4_, as well as the risk of H_2_ explosion in the method, limit its large-scale industrial application [34].

**Table 1:** Summary of physical and chemical methods for the synthesis of nZVI.

Preparation Methods		Process	Advantages	Disadvantages	Ref.
Physical methods	Vapor deposition method	The deposited material was gasified by physical means such as vacuum evaporation, ion plating, or sputtering to obtain nZVI powder by deposition.	High purity; small and uniform particle size	Complicated steps; high energy consumption; high cost	[35]
	Mechanical ball milling	The shear and impact forces provided by ball milling lead to the formation of various defects and changes in the crystal structure.	Simplicity; mild reaction conditions; solvent-free	Variations in one or more parameters can affect particle size; long cycle	[32]
	Ultrasound-assisted reduction	Under ultrasonic cavitation, gas-phase thermal reduction or liquid-phase reduction is carried out.	Smaller particle size; greater surface area; higher dispersity	Toxic byproducts	[36]
Chemical methods	Liquid-phase reduction	Mixing Fe^2+^/Fe^3+^ with NaBH_4_ solution to obtain Fe^0^ under a nitrogen atmosphere. Fe^2+^ + 2BH_4_^−^ + 6H_2_O → Fe^0^ + 2B(OH)_3_ + 7H_2_↑ 2Fe(H_2_O)^3+^ + 6BH_4_^−^ + 6H_2_O → 2Fe^0^ + 6B(OH)_3_ + 21H_2_↑	Apparatus and procedure simplicity; excellent reactivity and scalability	High cost; toxic byproducts	[13,37]
	Pyrolytic carbonyl iron process	Decomposition of Fe(CO)_5_ under high-temperature inert gas. Fe(CO)_5_ →Fe + 5CO↑	Cheap; easily available; adjust particle size with temperature	High energy; strict condition; high-toxic raw materials and byproducts	[38]
	Electrochemical method	Fe^2+^/Fe^3+^ in solution are reduced to nanoparticles at the cathode. Fe^3+^ +3e^−^ + stabilizer → nFe^0^	Simple; cheap; fast	Strong tendency toward aggregation	[39]
	Microemulsion method	In the presence of surfactant, Fe^2+^ was reduced by N_2_H_4_·H_2_O in a microemulsion system. At high pH: N_2_H_4_ − 4e^−^ + 4OH^−^ → N_2_↑ + 4H_2_O Fe^2+^ + 2e^−^ → Fe^0^	Simple device; low energy consumption; narrow particle size distribution; good interfacial property	High cost; secondary pollution	[33]
	Green synthesis	Biosynthesis of nanoparticles using plant extracts. Plant extracts + Fe^2+^ solution → Fe^0^	Low cost; environmental friendliness	Poor production; immature technology	[16]
	Gas-phase thermal reduction	Fe^2+^ is reduced to nZVI at elevated temperatures with the use of thermal energy in the presence of gaseous reducing agents.	Fewer byproducts	Strict conditions; dangerous, high temperature	[31]

Compared to classical methods, the green synthesis of nZVI has been proposed as a novel alternative and has become a prominent area of research in recent years. Studies have shown that the active components with high antioxidant capacity (e.g., polyphenols, free amino acids, and caffeine, among others) can be extracted from some natural plants such as pomegranate peel [40], green tea [30], oak, mulberry and eucalyptus leaves. These plant extracts can not only reduce Fe^2+^/Fe^3+^ to Fe^0^ but also act as effective capping and immobilization agents when synthesizing nanoparticles [41]. The synthesis, properties, and Cr(VI) removal performance of the green nZVI are summarized in Table 2. Among them, green tea extracts are the most appropriate reducing agents in green synthesis because they contain considerable amounts of polyphenols and caffeine substances. The preparation method of green nZVI is usually as follows: The washed natural plants were chopped or ground into powder after drying under chamber conditions or in an oven, added to deionized water, boiled for some time, and then vacuum filtered to obtain the green extract. Then, the Fe^2+^/Fe^3+^ solution is dropped, resulting in the formation of a black precipitate, which is nZVI. In general, the green synthesis method seems to have a better decontamination effect than the nZVI prepared by traditional methods, but it still has the following disadvantages: the solution has high acidity and salinity, which causes secondary pollution (chloride and acidification) and delivery (clogging due to Fe precipitation) when considering an in situ application of the reductant [42]. Furthermore, the output still could not meet the emerging requirement of the large-scale application.

**Table 2:** Summary of synthesis, properties, and Cr(VI) removal performance of the green nZVI.

Plants	Synthetic Method	Character	Cr(VI) Removal Performance	Ref.
*Thymus vulgaris* *Urtica dioica* * Rosa damascene*	Boiled the dried leaves of these plants at 80 °C for 1 h; added FeCl_2_·4H_2_O solution to the extract; adjusted pH to 6	The average size was 100 nm.	Over 90% Cr(VI) was removed with these three nZVIs for 10 min at an initial pH of 2 and a dose of 0.2 g/L.	[43]
Oak Mulberry Cherry leaf	Shook three kinds of dried leaf powders with water at 80 °C; mixed the leaf extract with 0.1M Fe (III) solution in a volume ratio of 3:1	Spherical and non-agglomerated nZVI with size within 10–30 nm.	CH-nZVI had a high adsorption capacity for Cr(VI) in the pH range of 4.0 to 8.0, up to 904.1 mg/g.	[44]
Green tea	Boiled 60 g/L leaves of dried green tea at 80 °C for 1 h; added 0.1 M FeSO_4_·7H_2_O solution to the extract in ratio of 2:3; adjusted pH to 6	Irregular shapes, varying sizes, and well dispersed with size within 40–60 nm.	Cr(VI) was completely removed in 18 min with the dosage of nZVI (0.7%) and acid (0.8 mol H^+^/kg chromite ore processing residues).	[45]
Mango peel	Boiled 12 g/L of the mango peel powder for 12 h at 80 °C; dropped the extract into 0.05M Fe (III) solution	--	Cr(VI) (95.45%) was removed in 10 min at the Cr(VI) concentration of 10 mg/L and a dose of 50 mg/L.	[46]
Barberry leaf	Boiled; filtered; dropped FeCl_3_ solution and adjusted pH	Shell-core structures with a diameter between 20 and 40 nm.	Cr(VI) (50 mg/L) was completely removed in 5 min at a dose of 0.675 g/L under photocatalysis.	[47]
Green tea (GT) Clove (CL) Spearmint (SM) Pomegranate (PG) Red Wine (RW)	Immersed leaves in water at 80 °C for 5 min while wine and pomegranate juice were used as received; mixed with 0.1 M Fe (III) solution at 300 rpm	Mainly spherical or elliptical with particle sizes of 50~60 nm, below 25 nm, not identified, 5~60 nm, below 30 nm, respectively.	The adsorption capacity of Cr(VI) was 500 mg/g at an initial pH of 2, higher than conventional nZVI.	[48]

Combining physical and chemical methods can reduce the BET surface area and size of nZVI and improve the materials’ activity to meet more demanding requirements. For example, the nZVI has been prepared by combining ultrasonic irradiation with gas-phase thermal reduction and liquid-phase reduction, respectively. The characterization analysis revealed a significant decrease in particle size and differences in particle morphology with the presence of ultrasonic cavitation compared to the sample obtained via synthesis without ultrasonic irradiation [36]. Graphite-encapsulated nZVI with higher purity and activity was synthesized by combining the ball milling method and low-pressure chemical vapor deposition [49]. nZVI with greater porosity and a larger specific surface area was prepared by combining ball milling, ultrasonic treatment, and alkaline leaching using pyrite and aluminum powder as raw materials [50]. In addition, some researchers found that, compared with nZVI particles synthesized at room temperature (the passivation layer thickness of 6~7 nm), the thickness of the passivation layer of nZVI particles synthesized under liquid nitrogen decreased to 3~4 nm [51].

## 3. Modified nZVI

Bare nZVI easily aggregates into chain-like structures due to its intrinsic magnetic properties, resulting in a decrease in its specific surface area, reactivity, and transport capacity [52]. Also, the surface passivation of nZVI particles blocks the electron transfer from Fe^0^, which reduces its reactivity and the removal performance of pollutants [53]. Therefore, addressing surface passivation and agglomeration is essential for further practical application of nZVI. Many technological methods, such as loading treatment, modification, and doping, were prospectively used to prepare composite nZVI-based systems.

### 3.1. Loaded nZVI

Using certain materials as carriers of nZVI, which generally have a large specific surface area and porous structure, can reduce the aggregation of nZVI particles and increase the contact area with pollutants. Common supporting materials include clay minerals, biochar, organic resins, reduced graphene oxide (rGO), etc. The preparation of loaded nZVI materials was mainly based on in situ liquid-phase reduction. Particularly, Fe(II) and Fe(III) are bound to the supporting material by electrostatic adsorption or cross-linking with functional groups and then reduced in situ by NaBH_4_. Table 3 summarizes the performance of nZVI-loaded materials for Cr(VI). nZVI-loaded materials showed better dispersion and activity than bare nZVI and, therefore, had a better Cr(VI) removal performance. Zhang et al. [54] synthesized silicon-rich attapulgite-nZVI, an environmentally friendly and efficient groundwater remediation material, which increased Cr(VI) removal efficiency by more than three times compared with silicon-poor attapulgite-nZVI. It is mainly attributed to the improved stability and dispersion of nZVI by Si-O-Fe coupling.

Bentonite-supported organosolv lignin-stabilized nZVI was synthesized with Cr(VI) removal efficiencies of 80–100% over a wide pH range (from 2 to 7) [55]. However, most studies have shown that the performance of nZVI for Cr(VI) removal from aqueous solution is greatly reduced under neutral and alkaline conditions. This limits the application of nZVI in the field of groundwater remediation. nZVI particles were successfully loaded on metal–organic framework materials/3D network porous polyacrylonitrile (3D MOF/PAN) film [56]. The removal efficiency of Cr(VI) was 20.54% higher than that of nZVI and remained above 82% after six regeneration cycles. However, when the pH value increased from 2.0 to 4.0, the Cr(VI) removal efficiency decreased by 51.8% due to the growth of the passivation layer under alkaline conditions, as well as the electrostatic repulsion with Cr(VI).

Biochar (BC) materials have been recognized as renewable carbon sorbents [57], attracting growing attention for their wide sources of raw materials, low cost, high porosity, and surface functional groups [31,58]. The adsorption and reduction of target pollutants can be achieved simultaneously by using biochar as the supporting material [59]. Previous research showed that organic components and silicate particles within biochar served as additional reaction sites of nZVI [60], and surface functional groups, such as C-O, O-H, aromatic C=C, aromatic C=O, and aromatic O-H, also interacted with Fe^3+^/Fe^0^ [61].

However, the use of original biochar-stabilized nZVI for pollutant removal remains limited due to its low reaction rate and the challenges associated with its regeneration [62]. To improve the performance of biochar as the nZVI particle carrier, researchers have carried out various chemical modifications on the original biochar [59]. For example, biochar modified with HCl, KOH, and H_2_O_2_ were used to support nZVI [63]. The characterization indicated that modification had little effect on the functional groups of the biochar surface, while the Brunner–Emmet–Teller (BET) surface area was increased to different degrees compared with the original biochar, which meant that a larger contact area was provided for nZVI and Cr(VI). Yi et al. [59] loaded nZVI onto phosphoric acid-modified biochar. The removal efficiency of Cr(VI) was significantly higher than that of P-BC and nZVI. Tian et al. [5] used polyethylenimide (PEI)-modified biochar as the carrier of sulfurized nZVI and found that PEI signally promoted the stability and reusability of nZVI and Cr(VI) removal rate. Another study showed that PBC could provide abundant amine groups and adsorption sites for heavy metals, and its adsorption capacity was much higher than that of the original biochar [64].

**Table 3:** Performance of loaded nZVI for Cr(VI).

nZVI Loaded Materials		Dosage /(g/L)	Cr(VI) Conc. /(mg/L)	pH	Equilibrium Time	Removal Rate	Ref.
Inorganic materials	Two-dimensional metal borides	0.1	10	2	-	82.4% (nZVI 69.6%)	[65]
	Attapulgite	2	40	6.2	30 min	91.63% (nZVI 48.68%)	[66]
	Bentonite	1.5	50	not adjusted	60 min	99% (nZVI 66%)	[67]
	Aminated magnesium hydroxide	0.5	10	3	5 min	97.92%	[68]
Carbon materials	Silicon-rich biochar	0.2	50	3.0	-	89.0% (nZVI 78.5%)	[69]
	AC	3	10	4.0	48 min	99.7%	[70]
	Layered double hydroxides (LDH)/rGO	1.8	10	<5	15 min	100%	[71]
	BC	1.25	100	5.6	18 h	100%	[72]
Organic materials	2,2,6,6-tetramethylpiperidine-1-oxyl (TEMPO) oxidized cellulose nanocrystal	0.5	20	7	60 min	99.96% (nZVI@carboxymethyl cellulose (CMC) 43.94%)	[73]
	Polydopamine-modified resin	0.05	100	5.0	60 min	94.5% (nZVI 33.4%)	[74]
	Lignin hydrogel	-	50	5.3	24 h	312.50 mg Cr^6+^/g Fe^0^ (11.6 times more than that of nZVI)	[75]

### 3.2. Surface Modified nZVI

Various stabilizers are coated on the surface of nZVI nowadays, including anionic or non-ionic surfactants, polymers, polyelectrolytes, and inorganic compounds, to keep nZVI in a stable and dispersed state. Table 4 summarizes the Cr(VI) removal performance of nZVI modified by different surface modifiers. Common stabilizing agents, such as chitosan, sodium alginate (SA), CMC, xanthan gum, Triton X-100 (TX-100), sodium dodecyl benzene sulfonate (SDBS), starch, etc., are available with functional groups such as carboxyl and amino groups on their surfaces [25]. These ionizable groups can promote the formation of covalent bonds between nZVI, which can increase the electrostatic and spatial repulsion between nZVI particles, thus limiting aggregation and enhancing the mobility of nZVI in porous media. In addition, the functional groups can form complexes with heavy metals, assisting in removing heavy metal ions.

Compared with the traditional nZVI and CMC-nZVI, SA-nZVI obtained by modifying nZVI with sodium alginate (SA) had better stability, dispersibility, and less sedimentation [22]. Pyrogallic acid (pyGA) was first introduced into nanoscale zero-valent iron (nZVI) synthesis [76]. TEM images revealed that compared with nZVI (40~90 nm), pyGA-nZVI presented a larger diameter of 110~140 nm, while the latter was characterized by low aggregation and high dispersion. The presence of sodium carboxymethyl cellulose (CMC) can increase Cr(VI) removal by both complexing precipitation with Cr(VI) compounds and improving the dispersion of nZVI in aqueous solution and porous media, while the excess amount of CMC reduces Cr(VI) removal by acting as a coating/shield on nZVI [52]. Dong et al. [24] selected starch, CMC, and SDBS, representing natural biopolymers, polyelectrolytes, and surfactants, respectively, to modify nZVI. It was found that stabilized nZVI was significantly better than bare nZVI in activating persulfate. Interestingly, the stabilization effect of SDBS on nZVI was more significant at low doses (SDBS/nZVI mass ratio of 0.1 wt%).

**Table 4:** Performance of surface-modified nZVI for Cr(VI).

nZVI Coated Materials	Dosage /(g/L)	Cr(VI) Conc. /(mg/L)	pH	Equilibrium Time/(min)	Removal Rate	Ref.
Zr^4+^ cross-linked chitosan	0.6	100	2.0	720	97.39% (nZVI 52.34%)	[77]
Starch	0.5	50	3	180	70% (nZVI 55%)	[78]
CMC	1.0	100	7.0	60	78.64% (nZVI ~50%)	[79]
Sodium alginate	0.015	4	6.0	20	96.4%	[22]
polyvinylpyrrolidone and sodium oleate	0.4	80	2.0	2	>99%	[80]
CaCO_3_	0.25	5	3.0	5	100% (nZVI 59.8%)	[81]

#### Controlled Release

In recent years, controlled release technology has attracted extensive attention in the remediation of groundwater pollution. Many researchers use controlled release materials (CRMs) to reduce the release rate of active components and extend the service life to improve the in situ repair effect. Iron leaching during redox reactions and the associated pollutants can cause secondary contamination to the aquatic environment, which may hinder the application feasibility of nanomaterials. Thus, for the nZVI system, another layer that can shield the release of dissolved Fe ions may be required.

In recent years, the use of hydroxide coating nZVI to achieve the purpose of slow release has attracted wide attention, such as Mg(OH)_2_-coated nZVI [82]. The novel Mg(OH)_2_ coating technique effectively improved the portability of nZVI and achieved the purpose of slow release. Moreover, nZVI nanoparticles modified with slow-releasing polymers as the matrix were also explored. The polymeric shell slowly releases nZVI into the groundwater, thereby acting as CRMs [83]. The nZVI/BC/CA composite was synthesized by capturing the biochar (BC) with nZVI in the calcium alginate (CA) bead matrix, which reduced the release of Fe while improving the removal activity. In the micro-electrolysis system, Fe species acting as the anode supplied electrons, and BC or CA acting as the cathode accelerated the redox reaction by mediating and transferring electrons to Cr(VI) without the need for direct contact between Fe^0^ and Cr(VI). Thus, the generated Fe^2+^ could be retained in the inner structure with a longer retention time, which created more reacting chances as Fe^2+^ anode, resulting in a better removal performance and more precipitation of iron oxides. In addition, the passivation of nZVI could be mitigated by reducing the direct contact among reactants and nZVI with BC or CA acting as the electron shuttles.

### 3.3. Bimetallic nZVI

Besides loaded nZVI and coated nZVI, researchers have demonstrated that the reactivity and reduction capacity of nZVI particles can also be enhanced through the deposition of a second metal catalyst onto the nZVI particles’ surface. The second metal, usually a noble or transition metal with a higher reduction potential [84], not only serves as the H_2_ carrier but also as a catalyst to accelerate the transformation of H_2_ into activated atomic H [85], thereby improving the performance of nZVI as an electron donor, including reduced passivation of Fe^0^ surface and accelerated reaction kinetics [86].

It is worth noting that the aggregation of bimetallic materials is still the major issue encountered in the process of synthesis and application [87]. In an attempt to reduce the aggregation of bimetallic particles, various porous supports and stabilizing agents have been utilized to stabilize and isolate the nanoscale bimetallic particles. The Cr(VI) removal performance and corresponding conditions of the modified nZVI bimetallic systems are summarized in Table 5. Currently, Fe/Ni, Fe/Cu, Fe/Pb, Fe/Ag, and Fe/Al are common bimetal systems. The previous works showed that the nZVI bimetallic system is more effective than nZVI in removing Cr(VI), and an efficient reduction of Cr(VI) can be achieved in the nZVI bimetallic systems within a very short time [88]. Considering the economic benefits and catalytic activity, Ni was often selected for research rather than noble metals, such as Ag and Pd. However, it should be noted that some of the second metals, including Ni and Cu, are toxic. Aluminum is considered an ideal material for preparing bimetallic nanomaterials because both the material and its oxidation products exhibit high environmental compatibility. nZVI bimetallic materials are mostly prepared by the borohydride reduction method in the liquid phase [86]. As this method may cause secondary contamination, the green synthesis of nZVI has been proposed as a novel alternative [89].

**Table 5:** Summary of the Cr(VI) removal performance of modified nZVI bimetallic systems.

Materials	Synthesis Methods	Dosage /(g/L)	pH	Cr(Ⅵ) Conc. /(mg/L)	Removal Efficiency	Ref.
Cu/Fe (Cu 3.0 wt%)	liquid phase co-reduction	0.1	-	100	68.94% (nZVI 37.47%) in 60 min	[90]
GT-nZVI/Cu	green synthesis method	0.4	5	5.0	94.7% (GT-nZVI 73.2%) in 60 min	[91]
Fe/Cu pre- and post-coated CMC	liquid-phase reduction	0.1	-	15	pH 5, CMCpost-Fe/Cu < CMCpre-Fe/Cu < Fe/Cu < Fe pH 9, CMCpost-Fe/Cu < Fe < CMCpre-Fe/Cu < Fe/Cu	[92]
Chitosan-stabilized Fe/Cu (Cu 1.0 wt%)	liquid-phase reduction	0.2	3–9	50	86.0–95.6%	[93]
nFe/Cu-loaded mesoporous silica	liquid-phase reduction	0.5	2	20	98.98% in 40 min	[94]
nFe/Cu-embedded cross-linked 3D hydrogel	self-assembly and in situ reduction	3.0	5	20	97.1% (nFe 54.6%) in 120 min	[95]
Santa Barbara Amorphous-15-supported Fe/Ni (Ni 10 wt%)	liquid-phase reduction	0.2	4	20	98.3% (nZVI 72.7%) in 60 min	[96]
Polyethylene glycol-stabilized Ni/Fe	liquid-phase reduction	0.6	5	50	>99% (nZVI 51.01%) in 60 min	[97]
Pd/Fe (Pd 1.0 wt%)	liquid-phase reduction	1.0	4	10	100% (nZVI 75%) in 1 min	[88]

## 4. Interaction Mechanism Between nZVI-Based Materials and Cr(VI)

Studying the interaction mechanism between nZVI-based materials and Cr(VI) can explain the reaction process, which is conducive to optimizing the adsorption/desorption conditions. It can improve the decontamination and regeneration performance of materials and has important significance in practical application. Previous studies have shown that the specific removal mechanisms involved in the treatment of heavy metal contamination with nZVI depend on the standard redox potential (E^0^) of the metal contaminant (Table 6). Specifically, metals with a more negative E^0^ than (or similar E^0^ to) Fe^0^ (e.g., Cd and Zn) are removed purely by adsorption onto the iron (hydr)oxide shell. E^0^ metals that are much more positive than those of Fe^0^ (e.g., Cr, As, Cu, and U) are preferentially removed by reduction and precipitation.

According to most current studies, the main interaction mechanisms could be regarded as adsorption, reduction, and co-precipitation [99,100]. Yirsaw et al. [46] investigated the mechanism of Cr(VI) removal by green synthesized nZVI and found that both Fe^0^ and Fe(II) were important components in reducing Cr(VI). Moreover, the presence of oxides and hydroxides of Cr(III) on the surface of GMP-nZVI indicated the strong binding of Cr(III) to GMP-nZVI. The Cr speciation on the surface of the S-nZVI particles after the reaction was characterized, which agreed with previous studies [101]: >95% of Cr existed in the reduced state Cr(III), including 70–75% hydroxide and 20–30% oxide. It is then speculated that the removal of Cr(VI) follows the above three-step mechanism.

### 4.1. Adsorption

The solid–liquid interfacial reactions, in which different forms of Cr(VI) (e.g., HCrO_4_^−^, CrO_4_^2−^, and Cr_2_O_7_^2−^) in solution are firstly adsorbed on the surfaces of nZVI, are the prerequisite for the following reduction and co-precipitation processes. The modification of carbon-based materials and various stabilizers makes the surface of nZVI-based materials rich in porous structures and oxygen-containing functional groups, providing abundant adsorption sites. In addition, the surface ferric hydroxide and oxide offer the coordinative and electrostatic functions to attract and adsorb charged ions, which enhance the adsorption capacity of heavy metal ions [39].

To understand the adsorption type of Cr(VI) removal by nZVI, different kinetic models and equilibrium adsorption isotherm models were utilized to interpret the uptake rate and equilibrium adsorption data of Cr(VI) by nZVI [84]. The experimental results showed that the valence state or electron transfer process rather than the boundary layer resistance of nZVI particles are the limiting factors affecting the chemical rate of Cr(VI) removal. Therefore, Cr(VI) removal by nZVI could be considered a single-layer chemisorption process. Wang et al. [102] explored the adsorption mechanism of Cr(VI) from aqueous solution by nZVI-modified dual surfactants (PS-nZVI) and obtained the consistent conclusion of chemisorption, including valence force produced by electron sharing or exchange between adsorbent and adsorbate. However, in Wei et al.’s study [103] on the removal mechanism of Cr(VI) in water by MnS encapsulating bagasse biochar-dispersed nZVI composite (MnS-nZVI@BC), about 40.89% of Cr(VI) was physically adsorbed onto the MnS-nZVI@BC by electrostatic attraction.

### 4.2. Reduction

The reduction process can be carried out through two pathways: direct reduction and indirect reduction. On the one hand, nZVI is an exceptional electron donor that can transubstantiate contaminants with a reduction potential (E^0^_H_) greater than −0.447 V [104]. Fe^0^ can be oxidized to Fe^2+^ at low pH (Equations (3) and (4)). A portion of Cr(VI) adsorbed on the surface of the nZVI-based material is reduced by obtaining electrons from the electron donor Fe^0^ (Equations (5)–(7)). Particularly, a bimetallic reduction potential difference leads to the second metal deposition on the nZVI surface of nZVI to form galvanic cells, which accelerate electron generation and transfer. On the other hand, they can also be adsorbed by the iron oxide shell of nZVI and then gradually reduced by Fe^2+^ derived from nZVI (Equations (8) and (9)). In the S-nZVI system, the random and disordered corrosion and dissolution of nZVI released excessive Fe(II) ions, which reacted with Cr(VI) in solution, resulting in an enhanced Cr(VI) removal and the generation of free radicals [105]. However, the core Fe^0^ would be covered by a dense FeS layer with the introduction of excess sulfur and constrained regeneration of Fe^2+^ [106].

The massive consumption of H^+^ in solution during the reduction process causes a rapid rise in pH. At last, the spontaneous precipitation of Cr(III) and the complexation between Cr(III) and Fe(III) occurred, leading to the generation of Cr(OH)_3_ (Equations (10) and (11)) or Cr(III)/Fe(III) hydroxides (Equations (12) and (13)) on the surfaces of nZVI-based materials.

Fe^0^ + 2H_2_O → Fe^2+^ +H_2_ + 2OH^−^ (3)

2Fe^0^ + 2H_2_O + O_2_ → 2Fe^2+^ + 4OH^−^ (4)

HCrO_4_^−^ + Fe^0^ + 7H^+^ → Fe^3+^ + Cr^3+^ + 4H_2_O (5)

2HCrO_4_^−^ + 3Fe^0^ + 14H^+^ → 3Fe^2+^ + 2Cr^3+^ + 8H_2_O (6)

Cr_2_O_7_^2−^ + 3Fe^0^ + 6H^+^ → 2Fe^2+^ + FeCr_2_O_4_ + 3H_2_O (7)

Cr_2_O_7_^2−^ + 6Fe^2+^ + 14H^+^ → 6Fe^3+^ + 2Cr^3+^ + 7H_2_O (8)

HCrO_4_^−^ + 3Fe^2+^ + 7H^+^ → 3Fe^3+^ + Cr^3+^ + 4H_2_O (9)

CrO_4_^2−^ + 3Fe^2+^ + 4OH^−^ + H_2_O → 3Fe(OH)_3_ + Cr(OH)_3_ (10)

Cr^3+^ + 3OH^−^ → Cr(OH)_3_ (11)

(1-x)Fe^3+^ + xCr^3+^ + 3H_2_O → Cr_x_Fe_1−x_(OH)_3_ + 3H^+^ (12)

(1-x)Fe^3+^ + xCr^3+^ + 2H_2_O → Cr_x_Fe_1−x_OOH + 3H^+^ (13)

### 4.3. Other Interaction Mechanisms

In various composite nZVI systems, the interaction mechanism becomes complicated due to different environmental conditions, possibly involving aggregation, oxidation, ion exchange, and other physical and chemical reactions. The carbon content decreased after the reaction of nZVI supported by ramie biochar(RBC-nZVI) with Cr(VI), indicating that RBC participated in the removal of Cr(VI) to a certain extent [107]. The possible interaction between Cr(VI) and RBC-nZVI is shown in Equations (14)–(16). At the same time, the changes in the content of C-C, C-O, C=O, O-C=O, and C-COOR further indicated that a complex reaction may have occurred. A research study implied that functional groups such as -OH, -COOH, and -C=O can be used as electron donors for Cr(VI) reduction and participate in Cr complexation [108].

3R-OH(s) + HCrO_4_^−^(aq) + 4H^+^ → 3R-O + Cr^3+^(aq) + 4H_2_O(l) (14)

3R-COOH(s) + Cr^3+^(aq) → (R-COO)_3_Cr(s) + 3H^+^(aq) (15)

R-C=O(s) + HCrO_4_^−^(aq) → R-C=O …HOCrO^3−^(s) (16)

In addition to concerns regarding the activity reactivity of nZVI-based materials, their longevity, fate, and migration in porous media, as well as their bioaccumulation and toxicity, must be taken into account for groundwater remediation. The early Cr(VI)-contaminated groundwater treatment technology was mainly ectopic treatment, namely extraction treatment technology, which was costly and time-consuming. It is worth noting that the associated process costs should be competitive with other existing technologies, so in situ processing technology, therefore, has gradually received attention. Abdul Mannan Zafar et al. [104] argued that the use of nZVI for groundwater contamination remediation is considered more sustainable than any known technology yet.

## 5. Application of nZVI in Remediation of Cr(VI)-Polluted Groundwater

In parallel to the research on the modification of nZVI on the laboratory scale, the application and monitoring of nZVI-based materials at contaminated sites, as well as the potential toxicity to organisms and the environment, are critical issues that must be addressed. nZVI is by far the most widely used nanoparticle (NP) in remediation [109]. nZVI-based materials have been shown to effectively remove Cr(VI) through direct groundwater injection techniques and permeable reaction wall (PRB) techniques.

### 5.1. Injection Well Technology

When nZVI is used for groundwater treatment, the injection well technology is the major method for in situ remediation. This technology is suitable for treating areas with extensive and severe pollution, offering the advantages of simple operation, short restoration period, low cost, and low disturbance. A pilot-scale in situ remediation test has been performed using commercially available nZVI in the Cr(VI) saturated zone of a historically tanning-contaminated site [110]. It was found that injection of nZVI resulted in a rapid immobilization of Cr(VI) without any substantial effect on the chemical properties of the groundwater. Meanwhile, the application of nZVI was demonstrated to have a positive effect on the autochthonous microflora regardless of possible concerns about nZVI ecotoxicity. However, transport of nZVI is normally limited by their aggregation and settling [111], which may result in a rapid loss in reactivity and limited transport distances, usually showing maximum transport distances of only 2.5 m [112] and 5 m in large containers and field trials, respectively.

Sulfidation of nZVI particles is a relatively new practice in the field of groundwater remediation, which gives more stability and enhanced electron transfer of nZVI. This method has been used in practical applications in removing chromium from groundwater [101], and the results showed that Cr(VI) concentration in groundwater decreased rapidly after S-nZVI injection. Moreover, S-nZVI was still active a few months after injection, indicating that S-nZVI can be efficiently used for Cr(VI) remediation at contaminated sites, given its good reactivity and longevity. For the field-scale synthesis of relatively large quantities of S-nZVI, it is recommended to optimize the S/Fe ratio for the intended application and mix the nZVI suspension continuously for about 1 h before adding dithionite and injecting it into the subsurface to minimize H_2_S formation during on-site synthesis [113].

Another option is to combine chemical reduction by nZVI and whey-supported biotic reduction to reduce and transform Cr(VI) into Cr(III) [114]. In this combined technology, complementarity exists between nZVI and the whey phases: nZVI can temporarily but rapidly reduce the Cr(VI) concentration, which not only prevents further spreading of the contamination but also has a generally positive impact on the autochthonous bacterial microflora in the aquifer soil. The application of whey promoted the development of anaerobic flora as an organic substrate and indirectly promoted the bioreduction of Cr(VI). Notably, the microbial populations also extensively reduced Fe(III) to the soluble Fe(II) form, resulting in an additional facilitation of Cr(VI) chemical reduction.

### 5.2. nZVI-Based Permeable Reactive Barriers (PRBs)

Permeable reactive barrier (PRB) technology is another commonly used method, excluding the injection well technology for groundwater in situ remediation. As the groundwater flows through the PRB, the pollutants are adsorbed, oxidized/reduced, immobilized, and degraded by the PRB reaction mediums to remove the pollutants. It is noteworthy that PRB has become more economically competitive for the in situ treatment of contaminated groundwater. The good environmental compatibility and strong chemical reducibility of nZVI allow it to serve as a reactive material in PRB, promising beneficial applications.

nZVI PRBs have been proven to be effective in removing Cr(VI) [115], U(VI) [116], cadmium [117,118], nitrates [119], phosphates [120], and nitramine compounds [121] from groundwater at a laboratory or field scale. Nevertheless, there are few literature reports on the application of nZVI PRBs to treat Cr(VI)-contaminated sites, while most laboratory-based research has been performed with configurations (e.g., packing column, batch and tank tests) and constant operating conditions (e.g., water composition, flow rate, temperature, and homogeneous reaction materials) [122], that differ from those in field-scale PRBs. The lack of field data on the use of nZVI for contaminated-site remediation contributes to a degree of uncertainty regarding the behavior, fate, and toxicity of nZVI particles.

According to current research, the main defects of nZVI PRBs are longevity reduction [123], device blockage, and environmental effects during the removal processes. The service longevity of the nZVI PRBs depends on the nZVI particle sizes and the physical and chemical properties of aquifers (e.g., hydraulic conductivity, pH, temperature, dissolved oxygen (DO), soluble metals, etc.). Henderson and Demond gathered operational data from several in situ PRBs from many pollution sites and identified that Fe^0^ reactivity was the main factor limiting PRB longevity compared with permeability reduction. The blockage of the nZVI PRB device usually occurs due to the formation of magnetite, ferrous hydroxide, and iron hydroxide precipitation, which reduces the permeability of PRB. At the same time, excavation of contaminated sites is operated in the process of PRBs, which not only requires a higher cost but exposes a large number of pollutants, resulting in a greater impact on the environment. It is usually suitable for aquifer environments with shallow buried depth (<20 m).

## 6. Impacts of nZVI-Based Particles on Living Organisms

Given the safety concerns associated with the potential negative effects of nanoparticle applications, conducting an environmental risk assessment of nanoparticles is crucial. To date, many influences of nZVI-based materials concerning living beings, like a few groups of microorganisms, plants, and animals, have been recognized at various levels. To better apply nZVI-based materials in practice, efforts should be made to answer three questions: What are the toxicity and impacts of nZVI particles on living organisms? How does the toxicity of nZVI change in aquatic environments? Can modification reduce the toxicity of nZVI?

### 6.1. What Are the Toxicity and Impacts of nZVI Particles on Living Organisms?

#### 6.1.1. Microorganisms

Some in vitro studies reported that nZVI had no cytotoxicity toward microorganisms. For example, nZVI exposure had no obvious effect on the survival and activity of the Gram-negative strain tested (*K. planticola*), even at the higher concentrations (10 mg/mL) after 24 h [124]. However, more studies reported that nZVI had a significant negative impact on the survival rate and biological activity of microorganisms. For example, nZVI was toxic to luminescent bacteria (*Photobacterium phosphereum T3*), and the toxicity increased significantly with the nanomaterial concentration. The addition of 500 mg/L of nZVI can reduce the relative bioluminescence intensity by 93.7% [125]. Studies have found that the toxicity induced by nZVI itself was limited, and the temporary negative effects are mainly attributed to the release of Fe^2+^ [126]. It has also been shown that complete inactivation of Escherichia coli at the initial concentration of 7.2 log CFU/mL is achieved after exposure to 0.1 g/L nZVI under anaerobic conditions for 30 min [127].

It was inferred that the virulence of nZVI to microbial populations may depend on the structure and dosage of the nanoparticles and the resistance of different microbial species. Some previous studies ascribe the cytotoxicity of nZVI to both the cell membrane disruption and oxidative damage induced by reactive oxygen species (ROS) [128]. Nonetheless, the mechanism of the intrinsic toxicity of nZVI to cells remains elusive. Therefore, more comprehensive and in-depth studies are needed to elucidate the mechanism of toxicity of nZVI.

#### 6.1.2. Plants

Iron has an active role in electron transport during respiration and photosynthesis. It is transported to the leaves to support photosynthesis, reproductive organs, and seeds to support embryogenesis. Available data suggest that nZVI can be used at low concentrations without adverse effects on plants and even enhance seed germination and plant growth, such as Typha latifolia (<50 mg/L) [129], Oryza sativa cv (~20 mg/L) [130], and peanut seedling (10–160 μmol/L) [131]. Nevertheless, nZVI exhibited a strong toxic effect on plants at higher concentrations, such as limiting stem growth of Typha, significantly reducing transpiration of hybrid poplars (>200 mg/L) [129], and inhibiting seed germination and seedling growth of mung bean (600~750 mg/L S-nZVI) [132]. It has also been shown that high concentrations of nZVI (200 mg/L) could significantly decrease the cytosolic Pi levels in Arabidopsis, causing low fresh biomass and weak chlorophyll fluorescence [133]. Additionally, the introduction of nZVI could shift the redox condition in the local environment and affect the oxygen release rate of plant roots [129].

In other words, the phytotoxicity of nZVI was dose-dependent, and the underlying mechanisms of nZVI toxicity to plants are unclear. Microscopic images indicated that a large amount of nZVI was coated on the plant root surface, which obstructed root membrane pores and interfered with water and nutrient uptake by plants [134]. This is one of the common mechanisms of phytotoxicity of nZVI-based materials. Although Fe nanoparticles accumulate in large quantities in the roots of plants, it is difficult for them to penetrate multiple layers of epidermal cells and transport upward from roots to leaves, indicating that nZVI has little effect on the aerial portion [132]. Oxidative stress damage caused by reactive oxygen species (ROS) may be another toxicity mechanism triggered by nZVI [135]. nZVI stimulates the production of reactive oxygen species (ROS) (H_2_O_2_, ∙OH, and ∙O_2_^−^), causing DNA damage and cell death, confirming the cytotoxicity and genotoxicity of ROS-mediated nZVI.

#### 6.1.3. Animals

Excess iron can be toxic to humans and other animals. The growing usage of nZVI in contaminated soil and water remediation has elicited large-scale environmental release, triggering the exposure of soil and aquatic animals. Limited studies have demonstrated a potential negative impact of nZVI on the growth and reproduction of earthworms [136,137] and the survival of Larvae of medaka fish survival [138]. CMC-nZVI was utilized at environmentally relevant concentrations (5–100 mg/L) for acute mortality and reproductive toxicity tests in *Caenorhabditis elegans* [139]. The results indicated that CMC-nZVI did not cause significant mortality in *C. elegans*. Nevertheless, CMC-nZVI parental exposure induced multigenerational reproductive toxicity in *C. elegans*, which can be ascribed to its high production of ROS in F0 (filial) generation, the toxicity of Fe(II)_aq_, and iron accumulation in *C. elegans*.

Moreover, a recent study reported that the presence of nZVI resulted in a high accumulation of Cr in the viscera of zebrafish instead of in the gills, revealing that nZVI—as a carrier of co-existing Cr—changes the main pathway of bioaccumulation in zebrafish [140]. In addition, 100 mg/L or more of nZVI injected into groundwater in applications accidentally released into nearby oxygen aquifers can be rapidly oxidized, which leads to acute hypoxia of aquatic organisms [141]. Overall, in vitro tests have identified that nZVI can be toxic to animal cell lines by producing reactive oxygen species, affecting bioaccumulation, and consuming oxygen.

The environmental release of nZVI caused during the production, transportation, application, and disposal of nZVI slurries can pose significant health and safety risks [109]. People working and living near nZVI manufacturing/application sites can uptake nZVI by skin contact, ingestion, or inhalation routes [142]. To date, a limited number of studies have evaluated the toxicity of nZVI in human cells. The effect of varying sizes of nZVI on genotoxicity in human peripheral blood cells was explored as the model in vitro system [142]. It was found that nZVI caused cytotoxicity by metabolic impairment and mitochondrial dysfunction, manifesting as acanthocytosis and spherocytosis, leading to hemolysis in erythrocytes, and was related to the size of nanomaterials. The genotoxicity induced by nZVI (~50 nm) can be attributed to its higher ion dissolution and colloidal destabilization. DNA damage by nZVI-1 (~15 nm) was possibly due to its smaller size and higher iron ion absorption.

### 6.2. How Does the Toxicity of nZVI Change in Aquatic Environments?

During the application of nZVI, its toxicity changes due to the constant changes in its physical structure and chemical composition (i.e., aging). In aquatic environments, nZVI can be oxidized to iron corrosion products, such as magnetite (Fe_3_O_4_), magnetite (γ-Fe_2_O_3_), and lepidocrocite (γ-FeOOH), via reacting with water and dissolved oxygen [143]. The corrosion rate and corrosion products of nZVI in water will vary with environmental conditions (e.g., temperature, iron type, dissolved oxygen concentration) and over time. Most studies have demonstrated that the toxicity of nZVI to *E. coli* gradually decreases with increasing aging time, possibly due to these iron (hydr)oxides that can reduce the adhesion of nanoparticles to microbial cells and inhibit electron transfer [143].

Li et al. [128] reported that this cell membrane damage caused by iron (hydr)oxide nanoparticles was insufficient to inactivate *E. coli* (less than 0.5 log inactivation). Fresh and aged nZVI and S-nZVI (aged 10 d, 20 d, and 30 d) were tested for toxicity in *E. coli* [144]. Similar to other studies, the toxicities of both nZVI and S/nZVI NPs were alleviated with aging time. The above studies demonstrate that the aging process leads to a significant reduction in the toxicity of nZVI-based nanoparticles to *E. coli*. Meanwhile, it was shown that nZVI at high concentrations was less toxic to Oryza sativa seedlings after 2 and 4 weeks of aging than fresh nZVI, but nZVI concentration and aging had no significant effect on Oryza Sativa germination [145]. Nevertheless, there are also studies based on nZVI toxicity in animals showing opposite results. For example, both nFe_3_O_4_ and Fe(II)_aq_ caused dose-dependent reproductive toxicity to a greater extent than CMC-nZVI in *Caenorhabditis elegans*, resulting in significantly decreased offspring [139]. Furthermore, CMC-nZVI and Fe^2+^-treated mature medaka pairs did not alter daily egg production (fecundity) or oxidative stress responses in observed tissues, while only nFe_3_O_4_ significantly decreased the fecundity of medaka among three iron species, with increased incidence of abnormal immature oocytes in the ovary [141].

However, most toxicity tests have been performed at batch scale under simplified solution conditions. Additional studies should be conducted to further assess the long-term toxicity of nZVI under more realistic conditions.

### 6.3. Can the Modification Reduce the Toxicity of nZVI?

In previous studies, larvae of medaka fish were thoroughly exposed to an aqueous solution containing CMC-stabilized nZVI and bare nZVI to learn the causal toxic effects of nZVI [138]. CMC-nZVI-mediated reaction led to a longer period of DO depletion and higher production of Fe (II)_aq_ and ROS compared to that of uncoated nZVI, which is one of the major causes of higher larval mortality with CMC-nZVI than nZVI. Aqueous hypoxia led to mass acute mortality of aquatic organisms. Redox-active Fe^2+^ has been found to result in the generation of active substances, such as ·OH. Excessive uptake of Fe (II)_aq_ would inhibit ion channels and oxidative stress, causing respiratory toxicity or neurotoxicity and contributing to the cell inactivation of nZVI.

Modified nZVI in subsequent studies could more or less alleviate the toxicity of nZVI. For instance, modifying nZVI with sodium alginate (a natural polysaccharide) and bentonite (a ubiquitous clay) not only decreased its aggregation/sedimentation and increased its transport, but both types of modified nZVI contained lower toxicities to Escherichia coli compared with bare nZVI [127]. Further studies found that modification with either alginate or bentonite can decrease the dissolution of Fe^2+^ from nZVI particles. Also, modified nZVI by biochar was less harmful to *E. coli* than nZVI.

Overall, by considering factors such as the actual environment, raw material availability, expected repair expectation, economic feasibility, and other factors, appropriate modification and supporting materials can be selected to modify nZVI and employ modified nZVI to remediate contaminated groundwater according to specific conditions [146].

## 7. Conclusions and Outlook

1. Based on the in-depth understanding of the characterization and physical and chemical properties of nZVI, the advantages and limitations of the application of nZVI in the field of environmental remediation have been thoroughly assessed. Aggregation and passivation of nZVI are widely recognized as the primary drawbacks that hinder its effectiveness in practical applications. Therefore, many studies have been carried out to address the challenges of nZVI through innovations in synthesis methodologies, exploration of modification methods, and enhancement of the material’s stability and mobility.
2. The preparation methods of nZVI (including mechanical ball milling, liquid phase reduction, green synthesis, etc.) and modification methods (such as loading, coating, doping, etc.) have been reviewed in this paper. The surface oxidation and agglomeration phenomena of the modified nZVI composite were significantly weakened, and its Cr(VI) removal performance was better than that of the original nZVI particles. Further, the combination of physical and chemical preparation methods or a variety of modification technologies can overcome the limitations of a single method and significantly enhance the Cr(VI) removal effect of the composite nZVI system. As a result, the development of multifunctional composite nZVI systems through the comprehensive application of diverse methods has garnered considerable attention in recent research.
3. The removal mechanism of Cr(VI) by nano zero-valent iron (nZVI) involves multiple processes, including adsorption, reduction, and co-precipitation. Additionally, in various composite nZVI systems, the interaction mechanism becomes complex due to different environmental conditions, which may involve physical and chemical reactions such as aggregation, oxidation, and ion exchange.
4. The excellent environmental compatibility and strong chemical reducibility of nZVI render it a promising reactive material for in situ remediation of Cr(VI)-contaminated groundwater, with significant potential for practical applications. However, due to cost and performance limitations, there are limited data on nZVI for on-site contaminated site remediation. Therefore, it is necessary to further optimize the process parameters, combine a variety of preparation and modification methods, and comprehensively consider the actual environmental conditions and the availability of raw materials to prepare a more stable, efficient, and cost-effective multifunctional nZVI composite material.
5. Most current toxicity test studies for nZVI have shown dose-dependence effects. However, a limited number of studies have found that the aging of nZVI enhances or reduces toxicity to organisms. Further research is required to more thoroughly evaluate the long-term toxicity of nZVI in real-world environmental conditions.

## Figures and Tables

**Table 6:** Standard redox potentials (E^0^) in aqueous solution at 25 °C [98].

Aqueous Solution	Half Reactions	E^0^ (V)
Chromium (Cr)	CrO_4_^2−^ + 8H^+^ + 3e^−^ → Cr^3+^ + 4H_2_O	1.51
Chromium (Cr)	Cr_2_O_7_^2−^ + 14H^+^ + 6e^−^ → 2Cr^3+^ + 7H_2_O	1.36
Arsenic (AsV)	H_3_AsO_4_ + 2H^+^ + 2e^−^ → HAsO_2_ + 4H_2_O	0.56
Copper (Cu)	Cu^2+^ + 2e^−^ → Cu	0.34
Uranium (U)	UO_2_^2+^ + 4H^+^ + 2e^−^ → U^4+^ + 2H_2_O	0.27
Iron (Fe)	Fe^2+^ + 2e^−^ → Fe	−0.44
Cadmium (Cd)	Cd^2+^ + 2e^−^ → Cd	−0.40
Zinc (Zn)	Zn^2+^ + 2e^−^ → Zn	−0.76

## Data Availability

No new data were created or analyzed in this study. Data sharing is not applicable to this article.
